# Identifying the Initiation of *Oshikatsu* and Health‐Promoting Activities Through *Oshikatsu* Among Older Adults: A Trajectory Equifinality Approach

**DOI:** 10.1111/ggi.70244

**Published:** 2025-12-10

**Authors:** Kengo Hosaka, Tomoki Tanaka, Katsuya Iijima, Misato Nihei

**Affiliations:** ^1^ Department of Human and Engineered Environmental Studies, Graduate School of Frontier Sciences The University of Tokyo Kashiwa Japan; ^2^ Institute of Gerontology The University of Tokyo Bunkyo‐ku Japan; ^3^ Institute for Future Initiatives The University of Tokyo Bunkyo‐ku Japan; ^4^ Department of Mechano‐Informatics, Graduate School of Information Science and Technology The University of Tokyo Bunkyo‐ku Japan

**Keywords:** frailty prevention, identification, *oshikatsu*, TEA, TEM diagram

## Abstract

*Oshikatsu*, supporting a specific individual or group, has been linked to enhanced well‐being and physical activity motivation. However, few studies have explored how this practice begins, particularly among older adults. This study applied the Trajectory Equifinality Approach (TEA) to investigate how individuals aged 50 and above initiate *oshikatsu* and subsequently engage in health‐promoting activities, and to identify influencing factors. Semi‐structured interviews were conducted with nine participants (men and women aged in their 50s–80s), and their narratives were analyzed using TEA. Six phases were identified: formative experience, encounter, event participation, social interaction, lifestyle improvement, and psychological fulfillment. Social enablers that facilitated these processes included relatives or friends who were fans, regular performances, venues close to home, understanding from family members, and disposable income. This study highlights that identifying with one's *oshi* serves as an intrinsic motivator for initiating health‐promoting activities.

## 1 Introduction

*Oshi* generally refers to a person or group—such as an idol or celebrity—whom an individual likes so much that they want to recommend them to others. The act of supporting them is referred to as *oshikatsu* [1]. The term gained broader recognition when it was nominated for the 2021 Buzzword of the Year Awards, a private prize in Japan [2]. To contextualize *oshikatsu*, it is helpful to compare it with related Western media concepts, such as “fandom” and “stan culture.” “Fandom” refers to communities of individuals with shared interests and personal identity expression [3, 4, 5]. In this sense, *oshikatsu* can be seen as a form of fandom activity. More recently, the term “stan” has emerged to describe an extremely devoted, sometimes obsessive, fan, and “stan culture” is often characterized by highly organized online activities [6].

While *oshikatsu* shares the communal aspects of fandom and the dedication seen in stan culture, its nuance lies in its link to personal well‐being and physical activity motivation. This is particularly relevant in later life, where *oshikatsu* may foster *ikigai* (a sense of life purpose) and vitality. As an academic topic, however, *oshikatsu* remains nascent, with few peer‐reviewed articles.

Meanwhile, in the fields of psychology and sociology, one‐sided intimate relationships with media figures are known as parasocial relationships [7, 8], and have received scholarly attention. Researchers have also highlighted the social and psychological significance of these relationships, including the formation of communities among individuals with shared interests and the expression of personal identity [9, 10]. Although the term *oshikatsu* is unique to Japan, similar practices have attracted interdisciplinary interest across various cultural contexts.

In the Japanese context, Inoue and Ueda [11] have noted the conceptual similarity between *oshikatsu* and psychological ownership. Psychological ownership is defined as a cognitive and emotional state in which an individual feels that an object—whether tangible or intangible—“belongs to them” [12]. Such objects are typically those to which people or groups feel strong attachment [13]. Inoue and Ueda [11] argue that psychological ownership in *oshikatsu* consists of two elements: a sense of psychological unity with the *oshi* and a sense of responsibility toward them. They found that when this sense of unity is strong, participants in *oshikatsu* tend to exhibit greater fellowship with other fans, higher levels of subjective well‐being, and stronger commitment to continuing the activity. These findings suggest that the fellowship fostered through *oshikatsu* may help prevent social isolation and enhance psychological well‐being.

There is also prior research that suggests the potential of *oshikatsu* from the standpoint of motivation. Tsuchiya et al. [14] propose a sports motivation model for regional revitalization that integrates *oshikatsu*, sports, and local resources. In a preliminary survey conducted for the same study, approximately 80% of university students reported having had the experience of taking action under the influence of their *oshi*, suggesting that *oshikatsu* can serve as an intrinsic motivator for a variety of behaviors, including physical activity.

This body of prior research indicates that *oshikatsu* is not merely a recreational activity, but a behavior with multifaceted significance, including psychological ownership, intrinsic motivation, social bonding, and enhanced well‐being. This is especially salient in later life, a period often marked by the loss of social roles and physical decline, which may lead to feelings of isolation [15, 16] and diminished motivation [17]. In this context, *oshikatsu* may serve as an opportunity to find *ikigai* and vitality in everyday life.

Most existing research on *oshikatsu* focuses on younger generations, with few studies targeting older adults. Furthermore, there are few studies that have identified in detail the experiences that lead to the initiation of *oshikatsu* and how the practice connects to health consciousness and activity. This study therefore focuses on individuals aged 50 and above—including both older adults and the so‐called “pre‐older adult” cohort—to explore how they begin *oshikatsu* and initiate health‐promoting activities through it, and how these practices bring about changes in their lives.

The purpose of this study is to identify the process by which individuals aged 50 and above who actively engage in *oshikatsu* come to initiate both the practice itself and related health‐promoting activities, as well as to clarify the factors that influence this process. Here, “health‐promoting activities” include behaviors supporting physical, cognitive, social, and emotional well‐being. To this end, the study addresses the following two research questions:

Research Question 1 *What kinds of experiences have older adults who actively engage in* *oshikatsu* *had prior to beginning the practice?*

Research Question 2 *What kinds of experiences have older adults who actively engage in* *oshikatsu* *had prior to initiating health‐promoting activities through the practice?*

## 2 Methods

In this study, the Trajectory Equifinality Approach (TEA) is adopted as the qualitative analytic method. TEA is characterized by the creation of Trajectory Equifinality Models (TEM), which visually and structurally organize individual experiences along a timeline in relation to social and institutional factors [18]. Whereas grounded theory aims at developing theoretical constructs, TEA is an interpretive and exploratory approach that seeks to capture both the commonalities and diversities of trajectories leading to a shared goal, the equifinality point (EFP). This feature makes TEA particularly suitable for understanding the diverse pathways through which older adults initiate *oshikatsu* and health‐promoting activities. To ensure transparency and completeness, the study is designed and reported in alignment with the COREQ checklist [19] to the extent possible.

As a concrete procedure, semi‐structured interviews were conducted with nine individuals (seven females, two males; seven individual interviews and one pair), aged 50 to 80, who had experience with *oshikatsu*. Participants described how they began oshikatsu, its link to health‐promoting activities, and perceived effects. Interview participants were recruited through the 2024 survey of the large‐scale Kashiwa Study on frailty, conducted by the Institute of Gerontology at the University of Tokyo [20, 21, 22]. Older adults who had demonstrated active engagement in fan‐support activities during a preliminary hearing at the survey venue were identified and invited to participate via face‐to‐face contact, email, or telephone. As a result, nine participants were recruited: Five older adults who were participants in the Kashiwa Study, two Kashiwa Study staff members who expressed interest in participating at the survey site, one acquaintance of a staff member who learned about the study and applied via email, and one individual previously known by the first author (Hosaka) to have engaged in *oshikatsu*, who was invited via email. The interviewer, Hosaka (male, 32), is a doctoral student with experience in service design interviews. He consulted with another researcher (Nihei) when developing the interview content. Except for one, he had no prior relationship with participants. All interviews included an explanation of the study and an ice‐breaking session. Each participant was interviewed twice (first session: questions; second session: feedback on the analysis and follow‐up questions), for a total of approximately 1.5 h in an individual interview format. However, depending on participants' preferences or health conditions, exceptions were made, such as conducting only one interview or holding a joint interview. Interviews were conducted online or at the Kashiwa‐no‐ha campus, depending on participant preference.

For the analysis, the interview data were transcribed verbatim and coded according to categories that contributed to the initiation of *oshikatsu* and health‐promoting activities: obligatory passage points (OPP), bifurcation points (BFP), social guidance (SG), social direction (SD), and EFP (2nd EFP). Based on these codes, each participant's experiences were then organized chronologically and visualized as a TEM diagram. The EFP was defined as “the initiation of *oshikatsu*”—regular engagement in support activities such as attending events. A second EFP (2nd EFP) was also defined as “the initiation of health‐promoting activities through *oshikatsu*.”

After creating individual TEM diagrams, an integrated TEM diagram was constructed by aligning the nine participants' common experiences in the same column and labeling them accordingly. SGs and SDs were consolidated wherever possible, while unique but important factors were also included. In the final diagram, actions taken by participants were represented with solid lines, whereas actions not taken were represented with dotted lines. The detailed definitions of the terms used in the TEM diagrams are presented in Table 1, and the symbols are shown in Table 2. The creation and integration of the TEM diagrams were conducted by Hosaka, who reported them together with the transcripts to Nihei. When interpretive differences arose between the two, revisions were made through discussion.

**TABLE 1:** Terminology of TEM diagrams.

#	Term	Definition	Application in this paper
1	Equifinality point (EFP)	A common action or state ultimately reached by participants.	Begins to regularly engage in active *oshikatsu*, such as attending events and concerts.
2	Polarized equifinality point (P‐EFP)	An opposing outcome that does not reach the equifinality point.	Does not come to regularly engage in active *oshikatsu*.
3	2nd equifinality point (2nd EFP)	A secondary action or state reached following the equifinality point.	Begins health activities through *oshikatsu*.
4	2nd polarized equifinality point (2nd P‐EFP)	An opposing outcome that does not reach the 2nd equifinality point.	Does not begin health activities through *oshikatsu*.
5	Obligatory passage point (OPP)	An event that is institutionally, habitually, or consequentially unavoidable for the participant.	Same as in the definition column.
6	Bifurcation point (BFP)	A point at which a choice regarding behavior emerged for the participant.	Same as in the definition column.
7	Social guidance (SG)	A force that encouraged choices toward the equifinality point.	Same as in the definition column.
8	Social direction (SD)	A force that hindered or inhibited the path toward the equifinality point.	Same as in the definition column.

**TABLE 2:** Symbols used in TEM diagrams.

EFP 2nd EFP	P‐EFP P‐2nd EFP	OPP	BFP	SG	SD	Realized actions	Unrealized actions
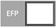	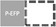	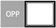	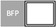	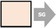	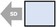	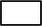	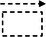

Unlike many qualitative approaches, the TEA method employed in this study emphasizes consistency by having a single researcher conduct both the interviews and analyses, while enhancing data reliability by confirming the accuracy of the TEM diagrams with the same participants across multiple interviews [18]. In addition, in this study, reliability was further strengthened through discussions of the TEM diagram content and interpretations among the researchers, thereby reducing potential bias.

This study was approved by the Research Ethics Committee of the University of Tokyo (approval no. 23‐47). Written informed consent was obtained from all participants prior to the interviews.

## 3 Results

**FIGURE 1:**
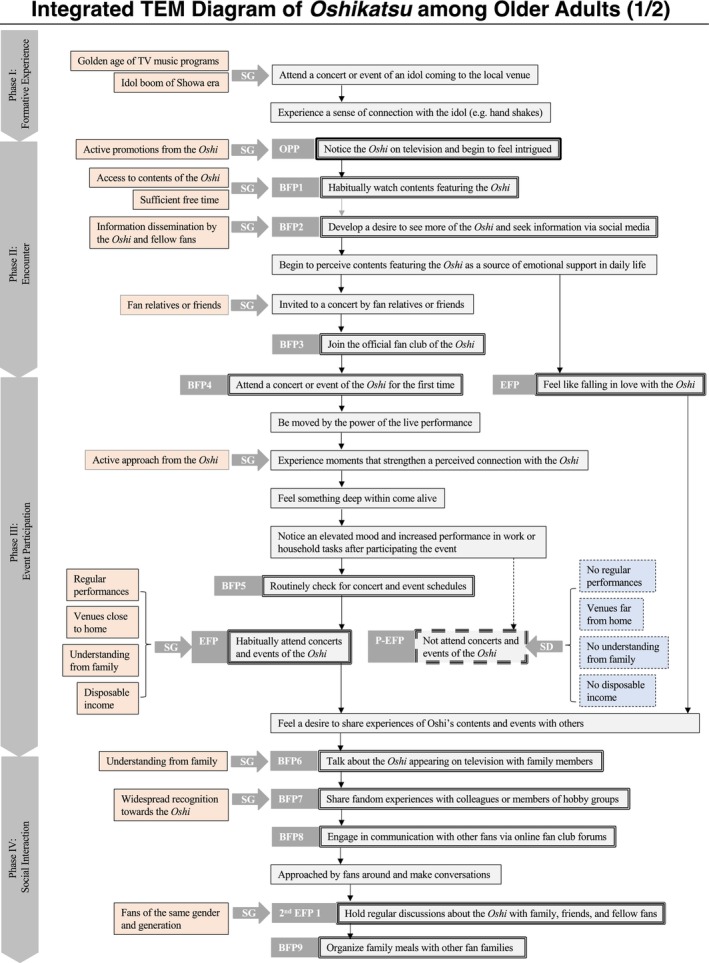
Integrated TEM diagram for the nine participants (1/2).

**FIGURE 2:**
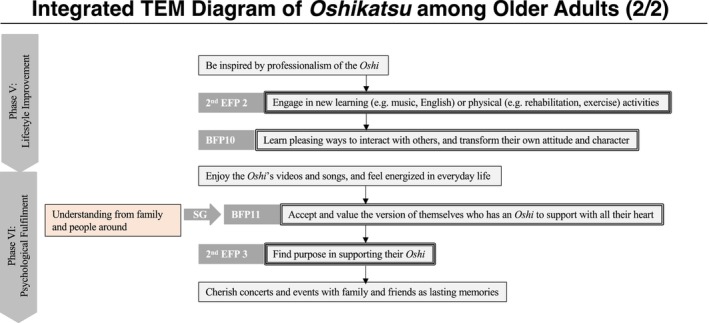
Integrated TEM diagram for the nine participants (2/2).

The profiles of the research participants, along with summaries of their interviews and narratives, are presented in Table 3. Additionally, a consolidated TEM diagram integrating each individual's diagram is shown in Figures 1 and 2. It was confirmed that older adults engaging in *oshikatsu* experience six phases in the process of initiating the practice and its development into health‐promoting activities.

**TABLE 3:** Summary of participants, interviews, and narratives.

Participant	Summary		
Ms. A.	Gender: female	Age: 87 years old	Care needs certification: not certified
	Residence: Kashiwa, Chiba		*Oshi*: Seiko Matsuda
	1st interview: Nov. 16, 2024 (70 min) with Mr. B		2nd interview: withdrew due to illness
	Ms. A became interested in Seiko Matsuda [23] after seeing her son, Mr. B, support the idol during his childhood (OPP). In later life, when invited by her son (SG), she began attending dinner shows together with him (BFP 4). Deeply moved by the atmosphere at these events, *oshikatsu* became a regular practice for her (EFP). Ms. A was influenced by Matsuda's cheerful personality, which contributed to the formation of a brighter and more communicative attitude toward others (BFP 10). As a result, she experienced increased family conversations (BFP 9) and more opportunities to go out and socialize.		
Mr. B.	Gender: male	Age: 54 years old	Care needs certification: not certified
	Residence: Kashiwa, Chiba		*Oshi*: Seiko Matsuda
	1st interview: Nov. 16, 2024 (70 min) with Ms. A		2nd interview: withdrew due to illness
	Mr. B first became interested in the female idol Seiko Matsuda during his junior high school years after seeing her on television (OPP), and he has supported her ever since. Through regular concert attendance (EFP), Mr. B, like Ms. A, was influenced by Matsuda's cheerful and positive personality, which brought changes to his own interpersonal behavior. For Mr. B, *oshikatsu* contributed to the formation of a more open and upbeat attitude toward others (BFP 10), which in turn resulted in increased opportunities for outings and social interaction (2nd EFP 1).		
Ms. C	Gender: female	Age: 64 years old	Care needs certification: not certified
	Residence: Ota, Tokyo		*Oshi*: Takuya Kimura
	1st interview: Nov. 17, 2024 (46 min)		2nd interview: May 24, 2025 (33 min)
	Ms. C first became interested in male idols during the Shōwa idol boom in her childhood (SG), when she supported Goro Noguchi [24]. After childrearing had settled down in adulthood (SG), she was drawn to male idol Takuya Kimura [25, 26] through his television dramas (OPP) and began attending concerts of his group, SMAP (EFP). For Ms. C, *oshikatsu* brought vitality and joy to her daily life, enhanced her performance in housework and employment, and fostered interaction with fellow fans (2nd EFP 1). Her activities were further encouraged by the influence of an aunt who also supported Kimura (SG).		
Ms. D	Gender: female	Age: 72 years old	Care needs certification: not certified
	Residence: Kashiwa, Chiba		*Oshi*: JIN (BTS)
	1st interview: Nov. 21, 2024 (47 min)		2nd interview: May 24, 2025 (34 min)
	Soon after the death of her husband, Ms. D happened to see JIN of the Korean boy band BTS [26] performing on a music program (OPP) and was captivated. She began to find enjoyment in everyday life through regularly watching Korean dramas (BFP 1) and gathering information of JIN daily via social media (BFP 2). After BTS was nominated for the Grammy Awards (SG), a world‐renowned music prize, she started openly identifying herself as a fan in the workplace and in hobby circles (BFP 7). Supporting JIN also led to increased outings and interaction with peers (2nd EFP 1), brought emotional healing, and inspired new dreams such as traveling to Korea. *Oshikatsu* thus became a catalyst for positive change in her life.		
Ms. E	Gender: female	Age: 68 years old	Care needs certification: not certified
	Residence: Kashiwa, Chiba		*Oshi*: Masaya Kamei
	1st interview: Nov. 23, 2024 (54 min)		2nd interview: June 8, 2025 (49 min)
	While attending classic music concerts as a retirement pastime, Ms. E encountered the pianist Masaya Kamei [27] (OPP). In order to attend his highly sought‐after live performances, she began regularly checking social media for concert information (BFP 2) shared by Kamei and his fans (SG). Her desire to share the excitement of these experiences with others led to increased social interaction (2nd EFP 1). Supporting the internationally active Kamei also motivated her to maintain her health and to begin studying English (2nd EFP 2), giving rise to new goals such as attending overseas performances. Furthermore, the presence of disposable income from monthly savings was identified as a social enabler (SG) that facilitated these activities.		
Ms. F	Gender: female	Age: 68 years old	Care needs certification: not certified
	Residence: Kashiwa, Chiba		*Oshi*: Satoshi Ōno
	1st interview: Nov. 24, 2024 (47 min)		2nd interview: May 26, 2025 (25 min)
	Encouraged by her son (SG), Ms. F, who had already recognized Satoshi Ōno of the male idol group Arashi [28] on television (OPP), began supporting him. Through streaming services (SG), listening to his music during housework became a regular routine (BFP 1). Over time, she began to feel that Ōno's music supported her daily life and even developed a sense akin to falling in love (EFP). For Ms. F, *oshikatsu* provided vitality in everyday life, increased family conversations (BFP 6), and fostered a more positive reappraisal of her daily routines (BFP 11). These activities were further supported by the understanding of her son and family toward her engagement (SG).		
Ms. G	Gender: female	Age: 61 years old	Care needs certification: not certified
	Residence: Kashiwa, Chiba		*Oshi*: Masatoshi Nakamura, Ryu Si‐won
	1st interview: Nov. 24, 2024 (73 min)		2nd interview: June 18, 2025 (34 min)
	Ms. G's engagement in support activities began in high school while watching high school baseball. She recognized the presence of Japanese actor Masatoshi Nakamura [29] through a television music program and Korean actor Ryu Si‐won [30] through a drama (OPP), which led her to start supporting them. She participated in concerts (EFP) and studied the Korean language as part of her *oshikatsu*. After joining a fan club (BFP 3), where many members were of her generation (SG), she also deepened exchanges with other fans she met through the fan club's online bulletin board (BFP 8). For Ms. G, *oshikatsu* became a motivation for going out and socializing (2nd EFP 1), as well as for language learning (2nd EFP 2), with her *oshi* serving as the driving force for these activities.		
Mr. H	Gender: male	Age: 76 years old	Care needs certification: not certified
	Residence: Kashiwa, Chiba		*Oshi*: Kashiwa Reysol
	1st interview: Nov. 30, 2024 (58 min)		2nd interview: June 1, 2025 (60 min)
	Mr. H first learned about the professional soccer club Kashiwa Reysol [31] on television (OPP), at a time when the newly founded team was actively promoted (SG). He began supporting the team, and several factors—including the weekly matches (SG), the proximity of the home stadium to his residence (SG), and the frequent opportunities for direct interaction with players in the early years (SG)—influenced his engagement, leading match‐watching to become habitual (EFP). Through these games, his connections with both fans and family deepened (2nd EFP 1). Even now, he regularly watches matches via streaming, with the team's wins and losses affecting his mood and appetite, bringing new vitality to his daily life. Moreover, supported by his family's understanding of his *oshikatsu* (SG), his activities contributed to a stronger sense of purpose in life (2nd EFP 3) and encouraged participation in local community activities.		
Ms. I	Gender: female	Age: 65 years old	Care needs certification: not certified
	Residence: Kumamoto, Kumamoto		*Oshi*: Y.M. (former member of Takarazuka Revue)^a^
	1st interview: Mar. 12, 2025 (53 min)		2nd interview: May 27, 2025 (22 min)
	Introduced by an acquaintance (SG), Ms. I first became aware of Y.M., an actress formerly affiliated with the [32] (OPP), and began supporting her. Through performances and fan club activities (BFP 3), she gained new energy in her daily life. Her fan engagement expanded to include out‐of‐town theater visits, travel, and participation in exercise classes organized by Y.M. (2nd EFP 2). These activities not only motivated her to maintain her health but also enhanced her enthusiasm for work.		

^a^ Following Y.M.'s retirement from Takarazuka Revue, her name is presented in initials at the request of participant Ms. I.

### 3.1 Phase I: Formative Experience

Three of the research participants had experiences before reaching adulthood in which they attended events for specific idols and felt a special connection with them. It was reported that SG factors—such as the “golden age of music programs” and the “idol boom”—played an influential role in shaping these experiences.

### 3.2 Phase II: Encounter

For seven participants, the encounter with their *oshi* occurred through television programs, with active promotion identified as a form of SG. Their interest deepened through regular viewing and further searches on social media. Through these behaviors, *oshikatsu* practitioners came to recognize that content featuring their *oshi* had become a source of support in their lives. Participants also reported that a spontaneous invitation from a relative or friend during this period often served as the catalyst for their first event attendance.

### 3.3 Phase III: Event Participation

Participants reported being overwhelmed by the impact of the first event they attended. At the same time, they experienced a psychological connection through their *oshi*'s active engagement during the event, resulting in a sense of inner revitalization that lingered well after it was over. This prompted them to regularly attend events. SG factors that supported this habituation included “regular scheduling of performances,” “proximity of venues to home,” “understanding from family,” and “financial flexibility.” Conversely, the absence of these conditions was identified as a form of SD.

### 3.4 Phase IV: Social Interaction

After experiencing Phase III and attending events regularly, participants began sharing their experiences with family and friends, motivated by a desire to convey the emotions they had felt. In this process, “High public recognition of their *oshi*” and “understanding from family” acted as SG. Moreover, as a result of this sharing, participants reported increased contact with other *oshikatsu* fans, leading to regular communication with family, friends, and other fans.

### 3.5 Phase V: Lifestyle Improvement

In Phase V, participants were influenced by their oshi's professionalism—on‐stage and off‐stage—and began engaging in self‐improvement activities such as studying music and English, building physical strength, and participating in rehabilitation. They also embraced their *oshi*'s value of bringing joy to others, shifting their attitudes and interpersonal behavior.

### 3.6 Phase VI: Psychological Fulfillment

**FIGURE 3:**
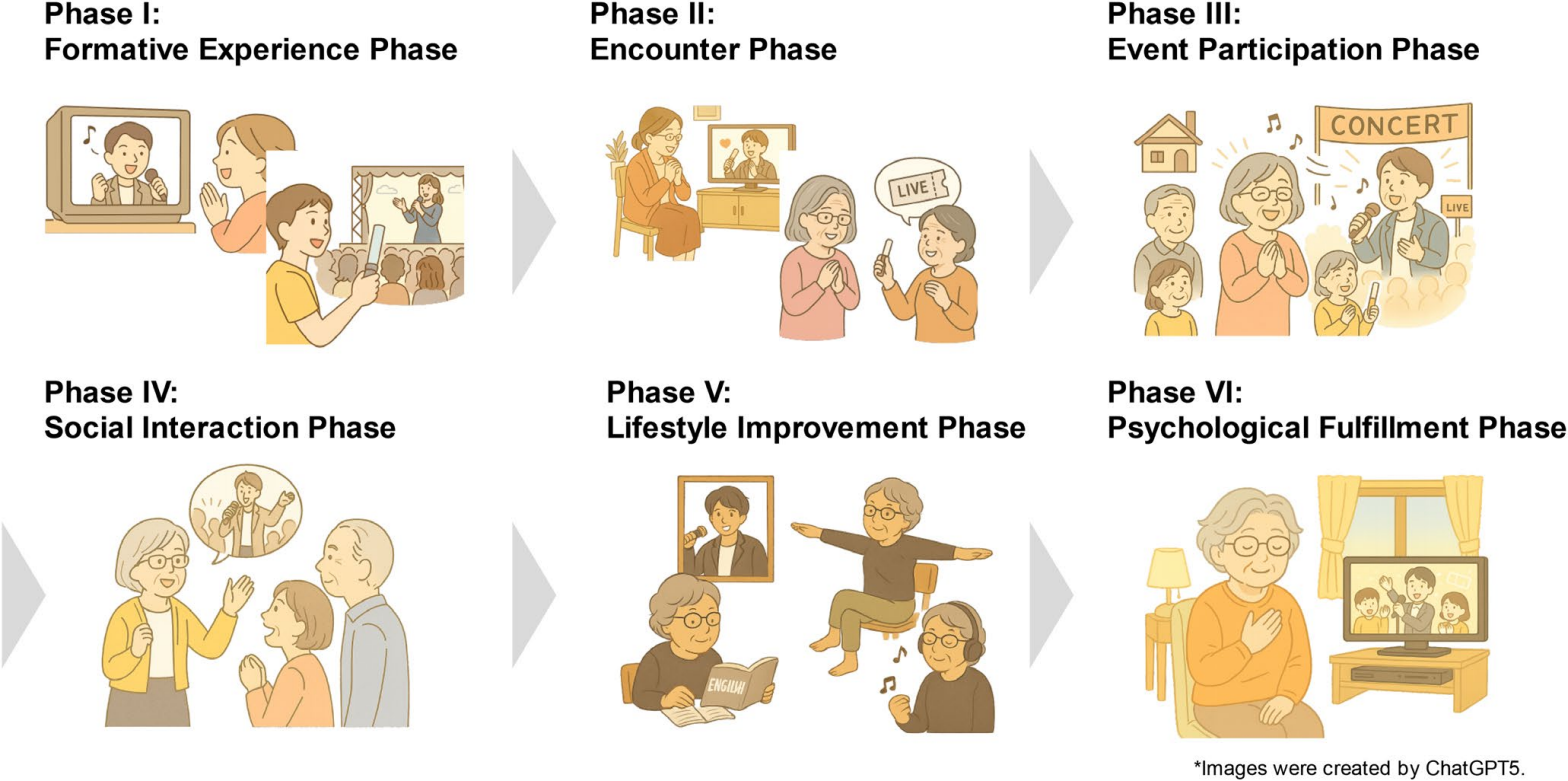
Conceptual image of older adults' *Oshikatsu* experiences.

In Phase VI, participants described feeling renewed vitality and enjoyment in their lives through continued engagement with content related to their *oshi*. Furthermore, understanding from family members and others toward their support activities functioned as SG, enabling them to view themselves more positively as individuals engaged in *oshikatsu*. As a result, they came to feel that *oshikatsu* had become their *ikigai* and it was confirmed that the memories of concerts and events shared with family and friends left a lasting impression on them. These six phases are illustrated in Figure 3.

### 3.7 Cumulative Trajectory Formation

In this study, the six phases leading to the initiation of *oshikatsu* (from Formative Experience to Psychological Fulfillment Phase) were commonly observed in participants' narratives. In addition, key elements such as “first‐time event attendance” serving as a major turning point, as well as social enablers—including family understanding, regular performances, venues close to home, and financial flexibility—were repeatedly confirmed across participants' accounts. These findings suggest a cumulative trajectory formation towards the initiation of *oshikatsu* and related health‐promoting activities.

## 4 Discussion

### 4.1 Pathways to the EFP (Initiation of *Oshikatsu* )

In relation to Research Question 1—the process by which older adults begin *oshikatsu*—the results of this study indicate that they reach the EFP (the initiation of *oshikatsu*) after passing through three key phases: Formative Experience Phase, Encounter Phase, and Event Participation Phase. Particularly noteworthy is the finding that “first‐time event attendance” serves as a major turning point toward continued engagement. The importance of this initial event suggests that it may function as an experience that extends beyond mere entertainment to exert a profound influence on participants' psychological states and social lives. While entering a new environment can pose psychological barriers for older adults, the presence of relatives or friends who are also fans—acting as SG—was found to play an important role in lowering those barriers. This underscores the value of real‐world interaction, unlike parasocial interaction [7].

Moreover, when the initial event leaves a strong impression, evokes emotion, or leads to self‐discovery, it can motivate older adults to integrate *oshikatsu* into daily life. SG factors that supported continued participation—such as “regular performances,” “venues close to home,” “family understanding,” and “disposable income”—reflect the practical and emotional needs of older adults. For example, “venues close to home” help ensure physical accessibility for those with mobility challenges; “understanding from family” provides emotional support, allowing individuals to enjoy the activity without psychological burden; and “disposable income” expands opportunities for leisure. Moreover, from the perspective of the *oshi*, the regularity of performances also emerged as an important factor. In fact, one participant in this study who had never experienced “first‐time event attendance” explained that the reason was the suspension of the *oshi*'s activities, further suggesting that the regularity of performances is a prerequisite for sustained engagement. When these conditions align, *oshikatsu* may become sustainable enjoyment, aligning with Stebbins' [33] “serious leisure.” This process contributes to the development of new social ties and to a sense of psychological fulfillment, including *ikigai* and *hari* (a sense of vitality or stimulation in daily life). These findings offer important insights from the perspective of promoting social participation and addressing social isolation in later life.

### 4.2 Pathways to the 2nd EFP (Initiation of Health‐Promoting Activities)

Regarding Research Question 2—how older adults initiate health‐promoting activities through *oshikatsu* (the 2nd EFP)—the psychological process of identification [34] was identified as playing a central role. This is a deep psychological mechanism in which an individual attempts to integrate the desirable traits or lifestyle of another person into their own identity. It also serves as the basis for the concept of modeling in Social Learning Theory [35]. Freud explained identification through the id (desire), ego, and superego: infants form an ego through interactions that balance desire and reality, and later internalize social values as the superego. A similar pattern appeared in older adults' *oshikatsu*. For example, Participant B's admiration for Seiko Matsuda (id) led her to adopt Matsuda's walking routine (ego) and embrace her cheerful persona as a personal ideal (superego). Participant E, inspired by Masaya Kamei (id), began studying English (ego) and developed a superego characterized by a proactive, challenge‐seeking mindset (superego).

Identifying with one's *oshi* as motivation for health‐promoting behavior is a key contribution of this study. Although Tsuchiya et al. [14] noted that *oshikatsu* may encourage sports participation, this study identifies identification as the core mechanism driving a broader range of health‐related activities among older adults. Unlike conventional health guidance, which can feel obligatory, *oshikatsu* is driven by positive emotions—desire to emulate one's *oshi* and enjoy the activity. This suggests a new approach to health maintenance that supports autonomy (sense of choice), competence (sense of capability), and relatedness (psychological connection with the *oshi*), in line with self‐determination theory [36]. Such motivation promotes spontaneity and sustainability, potentially leading to long‐term improvements in both physical and mental health. The integration of enjoyment and health behaviors through *oshikatsu* offers insights for extending healthy life and enhancing well‐being in older adults.

### 4.3 Potential Negative Aspects of *Oshikatsu* Among Older Adults

This study demonstrated the potential of *oshikatsu* to serve as a motivator for health‐promoting activities among older adults; however, its potential negative aspects must also be acknowledged. For older adults whose primary source of income is pensions, attending events and purchasing related goods may pose an economic burden, which in turn can exacerbate psychological strain such as worsening depressive symptoms [37]. Moreover, excessive support for a particular celebrity has been noted to carry addictive qualities, leading to a loss of psychological and social self‐regulation [38]. In addition, fan activities themselves may become stigmatized due to media portrayals, potentially causing friction with society and surrounding individuals [39]. Taken together, these considerations highlight that *oshikatsu*, while offering beneficial aspects for older adults, also constitutes a complex phenomenon that entails economic, psychological, and social risks. Future research should therefore aim for a comprehensive understanding that accounts for both its positive and potential negative dimensions.

### 4.4 Effectiveness of TEA in This Study

The use of TEA and TEM diagrams in this study carried several methodological advantages. First, TEA enabled the structuring of multiple experiences as diverse pathways leading to the EFP, thereby clarifying both the commonalities and differences in the processes through which *oshikatsu* was initiated and developed. Second, by visualizing BFP, SG, and SD within participants' narratives, the analysis facilitated an intuitive understanding of how social factors and individual behavioral choices were interconnected. Finally, due to the interpretive and exploratory nature of TEA, the phenomenon was not approached through a one‐directional theoretical construction by researchers, but was instead understood comparatively across multiple pathways. Based on these three points, TEA and TEM diagrams were considered highly appropriate for this study.

### 4.5 Limitations and Future Directions

This study elucidates the process through which *oshikatsu* begins and develops into health‐promoting activities; however, it has five limitations that should be considered in interpreting the findings. First, this is an exploratory study based on a qualitative approach, and the sample size is limited. Second, the use of convenience sampling may have introduced selection bias, making it more likely that participants with specific attributes or interests were included. Third, the health activities and psychological fulfillment identified in this study may not be unique to *oshikatsu*, but could also be observed in other forms of hobby activities. Fourth, some of the activities categorized as health‐promoting (e.g., language learning, music practice) may be more appropriately regarded as self‐improvement or cognitive engagement. However, given that such cognitive activities have been reported to contribute to the maintenance of cognitive function and reduction of dementia risk [40], they were treated as health‐promoting activities in this study. Fifth, the scope of theoretical saturation is limited. While the six phases related to the initiation of *oshikatsu* and health‐promoting activities, as well as the main facilitating factors, were consistently observed across multiple participants and judged to be saturated, the specific forms of health‐promoting activities were not fully captured. Therefore, caution is required when generalizing the findings to the broader population of older adults. Future studies should aim to complement and expand upon the present findings through the use of quantitative methods and analyses based on more diverse samples. Currently, a statistical analysis of health data comparing approximately 400 older adults with and without *oshikatsu* experience is underway.

## 5 Conclusion

This study used semi‐structured interviews with older adults and TEM diagrams to clarify how *oshikatsu* begins and leads to health‐promoting activities. Six phases were identified: Formative Experience, Encounter, Event Participation, Social Interaction, Lifestyle Improvement, and Psychological Fulfillment. Among these, first‐time event attendance had a particularly strong impact on initiation. Supportive guides (SG)—such as relatives and friends who are also fans—facilitated initiation, while regular performances, nearby venues, family understanding, and financial flexibility supported continued engagement.

Notably, *oshikatsu* offers not only physical and cognitive health benefits but also psychological stimulation and renewed vitality. The findings suggest that it helps older adults lead more active and fulfilling lives, supporting long‐term health. By emphasizing enjoyment—often overlooked in health programs—*oshikatsu* shows promise as a practical strategy for older adults.

It should be noted that the TEA employed in this study is an interpretive approach that organizes diverse pathways and meanings derived from participants' narratives. Accordingly, the findings of this study should not be regarded as direct empirical evidence that *oshikatsu* itself generates vitality or health behaviors among older adults, but rather as preliminary evidence indicating its potential. This serves as a foundation for future quantitative and interventional research.

At the same time, the generalizability of the findings is limited by the small sample size and the geographic concentration of participants. Future studies should aim to verify causal relationships between *oshikatsu* and health behaviors, and to explore the potential for interventional applications through a combination of quantitative research and longitudinal study designs.

## Conflicts of Interest

The authors declare no conflicts of interest.

## Supporting information


**Data S1:** ggi70244‐sup‐0001‐Supinfo.pdf.

## Data Availability

The data that support the findings of this study are available from the corresponding author upon reasonable request.
